# Near-field radiative heat transfer between high-temperature superconductors

**DOI:** 10.1038/s41598-020-73017-z

**Published:** 2020-09-30

**Authors:** S. G. Castillo-López, G. Pirruccio, C. Villarreal, R. Esquivel-Sirvent

**Affiliations:** Instituto de Física, Universidad Nacional Autónoma de México, Apartado Postal 20-364, Mexico, 01000 Mexico

**Keywords:** Nanoscale devices, Nanoscale materials

## Abstract

Near-field radiative heat transfer (NFRHT) management can be achieved using high-temperature superconductors. In this work, we present a theoretical study of the radiative heat transfer between two $\hbox {YBa}_2\hbox {Cu}_3\hbox {O}_{6.95}$ (YBCO) slabs in three different scenarios: Both slabs either in the normal or superconducting state, and only one of them below the superconductor critical temperature $T_c$. The radiative heat transfer is calculated using Rytov’s theory of fluctuating electrodynamics, while a two-fluid model describes the dielectric function of the superconducting materials. Our main result is the significant suppression of the NFRHT when one or both of the slabs are superconducting, which is explained in terms of the detailed balance of the charge carriers density together with the sudden reduction of the free electron scattering rate. A critical and unique feature affecting the radiative heat transfer between high-temperature superconductors is the large damping of the mid-infrared carriers which screens the surface plasmon excitation.

## Introduction

Superconductivity is relevant in a variety of fields ranging from high energy physics down to microwave technology. Loss-less conduction is attractive because it permits a device efficiency boost, as a consequence of the reduced noise, and lower power consumption. Superconducting materials are at the heart of several technologies, such as SQUIDs^1^, metamaterials^2,3^, nanowires of single-photon detectors^4^, among others. They are also suitable for creating qubits for quantum information^5–7^. Notably, for all those devices experiencing miniaturization, the proximity of several superconducting elements operated above or below their critical temperature deserves consideration. Closely packed superconducting elements may exhibit changes in their performances due to thermal coupling. The role of heat exchange via conduction in Josephson junctions^1,8^, ubiquitous in superconducting technology, has been recently investigated. On the other hand, thermal radiation between contactless superconducting materials may constitute the main heat channel, and thermal management has not been thoroughly investigated. High-$T_c$ films have been used as thermal radiation modulators in the far-infrared^9,10^, taking advantage of the large difference in the reflection and transmission between the normal and superconducting states. The measurements of transmission and reflection are used in these works to validate the phenomenological modeling of the dielectric function as a function of the temperature.

At submicron distances, radiative heat transfer is dominated by the evanescent modes supported by each material. Rytov fluctuation electrodynamics^11–13^ is adopted to treat the near-field radiative heat transfer. A large body of experimental and theoretical work exists^14–17^, including 2D-materials^18^, thin films^19–21^, polar dielectrics^22,23^, semiconductors^24–26^, composites^27–29^, layered structures^30^, hyperbolic materials^31^, and phase change materials^32–34^. The field of thermotronics^35^ deals with the shaping or control of NFRHT either by changing the dielectric properties of the materials through suitable combination of materials^36,37^, or using an external influence such as external magnetic fields^38,39^. The NFRHT strongly depends on the surface plasmon- and phonon-polaritons that are excited on the surface. Optimal conditions can be found to maximize the heat-transfer^40^ or through the hibridization of these surface modes^23,33,41^.

The purpose of this control is to envision possible applications as heat assisted recording^42^ or thermal transistors^43^.

Recently, suppression of the heat flux between parallel plates of BCS superconductors was shown^44,45^. This effect is seen at temperatures close to the absolute zero and at relatively large distances. However, the radiative properties of superconducting materials in the opposite range of temperatures and distances, particularly well below the Wien thermal wavelength, remain unexplored. In this context, the study of NFRHT between high-temperature superconductors (HTSCs) acquires prime relevance. Theoretical modeling of HTSCs optical response has been challenging since their discovery, and even today, consensus on several aspects has not been reached^46,47^. Important points of concern are: the pairing mechanisms, the pseudogap pairs forming even above $T_c$, the strange metal phase, and the phase coherence build-up^48^. The experimental techniques to investigate the physical properties of HTSCs includes optical measurements from the millimeter range to the ultraviolet, anisotropy and ellipsometry measurements, terahertz time-domain spectroscopy, DC conductivity and AC Hall effect. Expanding the possible experimental methods may contribute to understand the electrodynamics of these materials as well as other open problems in high-$T_c$ copper oxides superconductors (YBCO)^49^.

In this paper, we present a detailed analysis of the NFRHT between two HTSC slabs separated by a small vacuum gap in the range between 50 and 1000 nm. Our numerical calculations consider three different scenarios: Both YBCO plates are in the normal state, one plate is in the SC phase and the other is in the normal state, and both slabs are superconductors. We show a sharp drop in the total heat flux when the temperature of at least one of the slabs falls below $T_c$.

## Optical properties of YBCO

The physical properties of high-$T_c$ superconductors such as $\hbox {YBa}_{2}\hbox {Cu}_{3}\hbox {O}_{7-\delta }$ are strongly dependent on the oxygen deficiency given by the $\delta$ value. The crystalline structure is characterized by copper-oxygen planes, known as *ab*-planes, and copper-oxygen chains separated by yttrium atoms^47^. The direction normal to the *ab*-planes is called as the *c*-axis. The dielectric function of $\hbox {YBa}_2\hbox {Cu}_3\hbox {O}_{6.95}$ on the *ab*-plane has been measured by several authors for temperatures above and below $T_c$^47,48,50^. Similar measurements have been conducted along the *c*-axis^51,52^. Based on the experimental data, for temperatures above $T_c$, the dielectric function of $\hbox {YBa}_2\hbox {Cu}_3\hbox {O}_{6.95}$ on the *ab*-plane is described by a Drude contribution of free charge carriers plus an additional term in the mid-infrared frequencies (MIR) modeled by a Lorentz-type resonance. This contribution could be explained as electromagnetic absorption due to electronic interband transitions, or alternatively, to exciton formation^53^. In addition, there are also contributions from phonons described by Lorentz-type dielectric functions. The total *ab*-plane dielectric function reads

1 $$\begin{aligned} \varepsilon _n(\omega )=\varepsilon _{\infty }-\frac{\omega _{pn}^2(T)}{\omega ^2 + i \gamma _0 \omega } - \frac{\Omega _{mir}^2}{\omega ^2 -\omega _{mir}^2 +i \Gamma _{mir} \omega } -\sum _{l=1}^{6} \frac{S_l \omega _{ph,l}^2}{\omega ^2-\omega _{ph,l}^2+ i\gamma _{ph,l} \omega }. \end{aligned}$$

The parameters of the free charge carriers^50^ are $\omega _{pn}(100~\hbox {K})=11.4~\omega _0$, $\gamma _0=0.56~\omega _0$, the MIR parameters $\Omega _{mir}=39.5~\omega _0$, $\omega _{mir}=3.95~\omega _0$, $\Gamma _{mir} = 15.2~\omega _0$, $\varepsilon _\infty = 3.8$ and the phonon parameters $\omega _{ph,l}$, $\gamma _{ph,l}$, and $S_l$ are presented in Table 1. The plasma frequency in the normal state, but close to the critical temperature, is considered to have a constant value, $\omega _{pn}(100~\hbox {K})\equiv \omega _p$. Throughout this work, all the frequencies are normalized to $\omega _0=10^{14}$ rad/s. In the Supplementary information, Fig. S4, we show that the anisotropy induced by the *c*-axis has a negligible effect on the NFRHT between two semi-infinite YBCO plates.

In the superconducting state, we consider London’s two-fluid model of superconductivity^54^ which accounts for the basic electromagnetic properties of these materials, such as a perfect DC conductivity $\sigma _{DC} \rightarrow \infty$, the expulsion of weak magnetic fields from their interior (Meissner effect), and the formation of vortices under the action of an external magnetic field in type-II SCs. In the two-fluid model, the number (per unit volume) of carriers *n* is assumed to be constituted by a normal component of free charge carriers $n_n(T)$, with a finite damping rate $\gamma _0$, and a dissipationless superfluid component $n_s(T)$, *i*.*e*., $\gamma _0 \rightarrow 0$, so that $n=n_n(T)+n_s(T)$. It follows that $n_n(T_c)=n$ and $n_s(0)=n$. Taking into account that the electronic supercurrent is transported by $n_s/2$ Cooper-like pairs with charge 2*e* (and mass 2*m*), then the current density may be expressed as $j=j_n + j_s =n_n e v_n + (n_s/2)(2 e) v_s$, where $v_n$ and $v_s$ are the respective mean velocities. Clearly, for $T>T_c$ the total current density is normal, while at $T=0$ all of the current is superfluid; in the intermediate range $T_c> T > 0$ it involves partial contributions. However, in this regime the conductivity of the system is infinite: by assuming that each current density is transported with respective conductivities $\sigma _n$ and $\sigma _s$, then the total parallel conductivity is $\sigma _t=\sigma _n + \sigma _s$. In the limit $\sigma _s \rightarrow \infty$ then $\sigma _t = \sigma _{DC} \rightarrow \infty$.

In the SC state, the fraction of condensed electrons does not show dissipative scattering, so that $\gamma _0 \rightarrow 0$. In that limit, $\left( \omega \pm i \gamma _0 \right) ^{-1} \rightarrow \mathcal{P} \left( 1/\omega \right) \mp i \pi \delta (\omega )$, and the dielectric function becomes:

2 $$\begin{aligned} \varepsilon _{s}(\omega )=\varepsilon _{\infty }+\frac{ i \pi \omega _{ps}^2(T)}{2 \omega } \delta (\omega )-\frac{\omega _{ps}^2(T)}{\omega ^2 }-\frac{\omega _{pn}^2(T)}{\omega ^2+i\gamma _0\omega }- \frac{\Omega _{mir}^2}{\omega ^2 -\omega _{mir}^2 + i\Gamma _{mir} \omega } -\sum _{l=1}^{6} \frac{S_l \omega _{ph,l}^2}{\omega ^2-\omega _{ph,l}^2+ i\gamma _{ph,l} \omega }. \end{aligned}$$

In this case $\omega _{ps}(2 \mathrm { K})=11.4~\omega _0$, while the parameters $\Omega _{mir}$, $\omega _{mir}$, $\Gamma _{mir}$ and $\varepsilon _\infty$ are the same as those obtained in the normal situation. The phonon parameters shown in Table 1 are also very similar. It is expected that the plasma frequency of the superfluid component does not vary for temperatures $T \le 2$ K. The fact that $\omega _{ps}(2~\hbox {K})= \omega _{pn}(100~\hbox {K})=\omega _p$ indicates that the dielectric response of this system is consistent with the two-fluid model. Notice that the MIR contribution involves a large oscillator strength $(\Omega _{mir})$ and since $\Gamma _{mir} = 15.2~\omega _0$, the Lorentzian extends over a wide frequency range down to the low frequency. The dielectric function, Eqs. (1)-(2), is in good agreement with the experimental observations^48,50^. Depending on the oxidation of the compound (given by $\delta$) it is posible to have a full metallic behavior that screens the phononic contribution^55^. In our case, the term in the dielectric function corresponding to MIR will not affect the phonons at lower frequencies.

**Table 1:** Parameters of dielectric function of $\hbox {YBa}_2\hbox {Cu}_3\hbox {O}_{7-\delta }$ in normal ($T = 100$ K) and superconducting ($T = 2$ K) states normalized to $\omega _0=10^{14}$ rad/s^50^.

*l*	$T=100$ K			$T=2$ K		
	$\omega _{ph,l}/\omega _0$	$\gamma _{ph,l}/\omega _0$	$S_{l}$	$\omega _{ph,l}/\omega _0$	$\gamma _{ph,l}/\omega _0$	$S_{l}$
1	0.29	$5.8\times 10^{-3}$	31	0.29	$4.5\times 10^{-3}$	31
2	0.36	$1.8\times 10^{-2}$	3	0.36	$5.0\times 10^{-3}$	2
3	0.52	$3.4\times 10^{-2}$	6	0.52	$3.9\times 10^{-2}$	10
4	0.59	$2.0\times 10^{-2}$	10	0.58	$1.7\times 10^{-2}$	12
5	1.00	$1.0\times 10^{-1}$	2	1.00	$9.4\times 10^{-2}$	3
6	1.07	$3.0\times 10^{-2}$	2	1.06	$3.2\times 10^{-2}$	2

In order to perform a detailed characterization of the thermal properties of the system it is necessary to construct a model of $\varepsilon (\omega )$ in a wide temperature interval including the extremal values involved in the experimental studies mentioned above. For that purpose we assume that their charge transport properties may be described in terms of a 2D gas of weakly-interacting fermion pairs that condense at $T_c$. These compound particles define a dilute gas in YBCO, since the mean inter-pair distance $(l_0)$ largely exceeds the pair size defined by the correlation length $(\xi _0)$, *i.e.*, $l_0 \sim 7 \xi _0$^56^. On these grounds, the dynamics of HTSCs charge carriers may be accounted by means of a weakly-interacting Bose gas embracing elementary excitations with a Bogoliubov spectrum ${\mathscr {E}}(p)=\left( p^2 c_s^2+(p^2/2m)^2 \right) ^{1/2}$ (with $c_s$ the sound speed and *p* the boson momentum); in the low-momentum limit, this yields a phonon spectrum, ${\mathscr{E}}(p) \rightarrow c_s p$. It is straightforward to show^57^ that a 2D gas with a linear spectrum satisfies:

3 $$\begin{aligned} \frac{\omega _{ps}^2(T)}{\omega _{ps}^2(0)} =\frac{\lambda _{L}^{-2}(T)}{\lambda _{L}^{-2}(0)}=1-\left( \frac{T}{T_c}\right) ^2. \end{aligned}$$

This relation gives an accurate representation of experimental measurements of the London penetration length $\lambda _L(T)$ in the $\hbox {CuO}_2$ plane for a wide range of dopings of $\hbox {YBa}_2\hbox {Cu}_3\hbox {O}_{7-\delta }$ samples, as well as of the dependence of the transition temperature on doping^58^. Consistent with Eq. (3), the plasma frequency of the normal component of free charge carriers is given by the temperature-dependent function

4 $$\begin{aligned} \frac{\omega _{pn}^2(T)}{\omega _p^2}=\left( \frac{T}{T_c}\right) ^2, \qquad \hbox {for}\quad T<T_c. \end{aligned}$$

**Figure 1:**
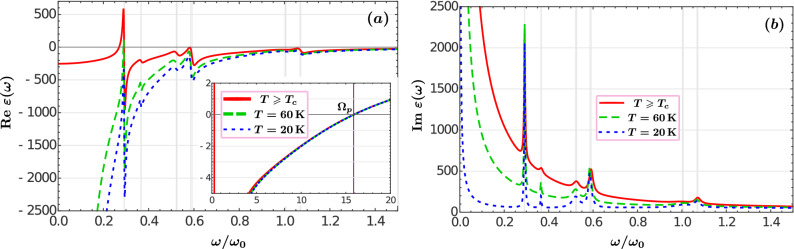
Frequency dependence of the real (**a**) and the imaginary (**b**) parts of the YBCO dielectric function for three different temperatures, Eqs. (1)–(2). The inset is a close up around the effective plasma frequency $\Omega _p$. The horizontal axis is normalized to $\omega _0=10^{14}$ rad/s.

In the following we analyze the NFRHT induced by the optical response described by Eqs. (1)–(4) for the temperature-dependent $\hbox {YBa}_2\hbox {Cu}_3\hbox {O}_{6.95}$ dielectric function. Figure 1 shows the permittivity of $\hbox {YBa}_2\hbox {Cu}_3\hbox {O}_{6.95}$ as a function of the frequency for three different temperatures (indicated in the figure). In Fig. 1a the real part of the dielectric function exhibits large negative values in the low frequency region for $T<T_c$, consistent with a metallic behavior. As the temperature decreases, the real part of the permittivity becomes more negative and its imaginary part drastically diminishes, Fig. 1b, meaning that the YBCO plates become highly reflective. The zero of the real part of the dielectric function, Eqs. (1)–(2), shows that there exists a bulk plasmon around $\Omega _p = 16~\omega _0$ in agreement with experimental evidence^50^, see inset of Fig. 1. An approximate estimation of this parameter by Tanner and Timusk^46^ using a non-dissipative two-component model yields

5 $$\begin{aligned} \Omega _p\approx \left[ \left( \omega _{p}^2+\Omega _{mir}^2\right) /\varepsilon _{\infty }\right] ^{1/2}\approx 21\omega _0. \end{aligned}$$

However, both free charge carriers and MIR electrons have a finite dissipation given by $\gamma _0$ and $\Gamma _{mir}$ parameters, respectively, shifting the effective plasma frequency, $\Omega _p$, to lower values. As the temperature decreases below $T_c$, dissipation channels provided by the normal component of free charge carriers diminish. Nevertheless, because of the large damping rate $\Gamma _{mir}$ of MIR electrons, this effect is negligible, and the effective plasma frequency is practically temperature independent.

**Figure 2:**
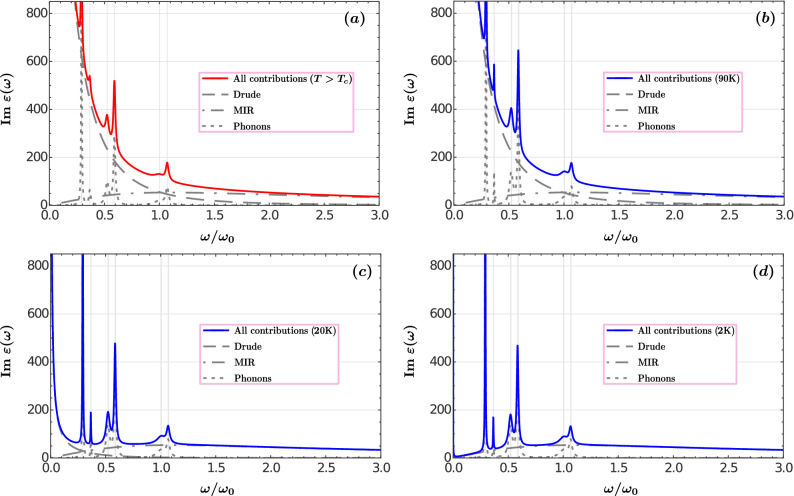
Individual contributions of the Drude (dashed line), the mid-infrared (dash-dotted line) and the phonons (dotted line) bands to the imaginary part of the normal (**a**) and superconducting (**b**–**d**) $\hbox {YBa}_2\hbox {Cu}_3\hbox {O}_{6.95}$ dielectric function.

Figure 2 shows the individual contributions of the Drude, mid-infrared, and phonon bands to the imaginary part of $\hbox {YBa}_2\hbox {Cu}_3\hbox {O}_{6.95}$ dielectric function (1)–(2). The sharp resonances of the different phonon contributions to the dielectric function are depicted by the dotted-line curve, while the dashed and dash-dotted smooth curves represent the Drude and mid-infrared terms, respectively. The complete dielectric function of the normal YBCO is presented in Fig. 2a with a solid red line. Notice that within the phonon frequency range, the optical response is mainly governed by the Drude contribution of free charge carriers. The imaginary part of the permittivity at higher frequencies ($\geqslant 2\omega _0$) is practically the same as that only due to the MIR band of bound electrons. When the temperature decreases below the superconducting transition $T<T_c$, the density of non-dissipative electron pairs, $n_s(T)$, increases, while the normal component of the free charge carriers density, $n_n(T)$, decreases. This implies that the electron damping mechanism within the Drude term is suppressed at finite frequencies and uncovers the strong participation of the temperature-independent mid-infrared band in the imaginary part of the $\hbox {YBa}_2\hbox {Cu}_3\hbox {O}_{6.95}$ permittivity, see Fig. 2b–d. For the individual contributions of the Drude, MIR, and phonon bands to the real part of YBCO permittivity see Supplementary Information, Fig. S1. It shows the frequency range within which each band participates in the plasmonic-like response of the material.

## Radiative heat transfer

**Figure 3:**
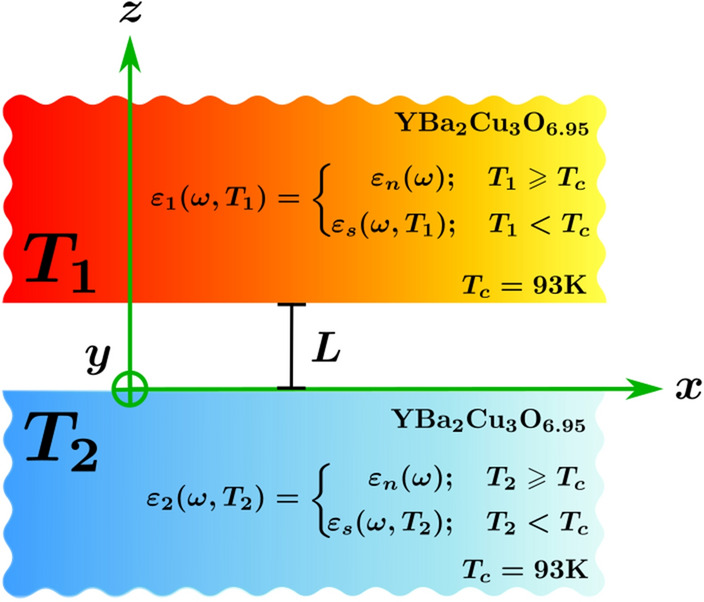
The system under study consists of two parallel slabs of $\hbox {YBa}_2\hbox {Cu}_3\hbox {O}_{6.95}$ each with a temperature-dependent dielectric function.

Our system consists of two slabs of a high-temperature superconducting material separated by a vacuum gap of length *L*, as depicted in Fig. 3. One slab is kept at temperature $T_1$, while the other at temperature $T_2$, with $T_1\ne T_2$. Each plate is described by a local dielectric function $\varepsilon _1(\omega ,T_1)$ and $\varepsilon _2(\omega ,T_2)$. NFRHT originates from thermally excited electromagnetic fields within the materials^13^. The theory developed by Rytov has the advantage that, for parallel plates, the physical consequences of the fluctuating fields are expressed in terms of the reflection coefficients for P and S polarized waves, and the radiative heat transfer between the two plates has contributions from both propagating and evanescent electromagnetic waves. When the separation between the plates is less than the Wien thermal wavelength, i.e., $L\le \lambda _T$, the contribution of the evanescent waves dominates and the total radiative heat flux between the plates exceeds the Stefan–Boltzmann prediction for black bodies. Another characteristic of the NFRHT is that it depends on the separation between the slabs. The total heat flux is

6 $$\begin{aligned} Q_T(L,T_1,T_2)=\int _{0}^{\infty }d \omega ~ S_{\omega }(\omega , L,T_1,T_2), \end{aligned}$$

where $S_\omega$ is the spectral heat flux given by,

7 $$\begin{aligned} S_{\omega }(\omega ,L,T_1,T_2)=\left[ \Theta \left( \omega ,T_1\right) -\Theta \left( \omega ,T_2\right) \right] \sum _{j=p,s}\int \frac{d\beta \beta }{(2\pi )^2}\left[ \tau _j^{\mathrm{prop}} \left( \omega ,\kappa ,L\right) +\tau _j^{\mathrm{evan}}\left( \omega ,\kappa ,L\right) \right] . \end{aligned}$$

Here $\Theta \left( \omega ,T\right) =\hbar \omega /\left[ \exp \left( \hbar \omega /k_B T\right) -1\right]$ is the average energy of the Planck oscillator at the angular frequency $\omega$. The components of the wave vector parallel to the interfaces, $\beta$, and normal to them, $\kappa$, are related to each other as $\kappa =\sqrt{\omega ^2/c^2-\beta ^2}$ inside the vacuum gap, and $\kappa _i=\sqrt{\varepsilon _{i}\omega ^2/c^2-\beta ^2}$ within the medium characterized by a dielectric function $\varepsilon _{i}$ with $i=1,2$.

In Eq. (7), the sum considers the contribution of both P and S electromagnetic waves, via their transmission coefficients $\tau _p$ and $\tau _s$. These coefficients are expressed differently for propagating ($\beta <\omega /c$) and evanescent ($\beta >\omega /c$) waves, as follows

8 $$\begin{aligned} \tau _{j=p,s}^{\mathrm{prop}}\left( \omega ,\kappa ,L\right) =\frac{\left( 1-|r_{j}^{(1)}|^2\right) \left( 1-|r_{j}^{(2)}|^2 \right) }{|1-r_{j}^{(1)}r_{j}^{(2)}\exp \left( 2i\kappa L\right) |^2}\qquad \hbox {and} \qquad \tau _{j=p,s}^{\mathrm{evan}}\left( \omega ,\kappa ,L\right) =\frac{4\hbox {Im}(r_{j}^{(1)})\hbox {Im}(r_{j}^{(2)}) \exp \left( -2|\kappa |L\right) }{|1-r_{j}^{(1)}r_{j}^{(2)} \exp \left( -2|\kappa |L\right) |^2}, \end{aligned}$$

where $r_{j}^{(i)}$ is the Fresnel reflection coefficient at the interface between the *i*-half space and the vacuum. It read as $r_{p}^{(i)}=(\varepsilon _{i}\kappa -\kappa _i)/(\varepsilon _{i}\kappa +\kappa _i)$ for P polarization, and $r_{s}^{(i)}=(\kappa -\kappa _i)/(\kappa +\kappa _i)$ for S polarization.

## Results

**Figure 4:**
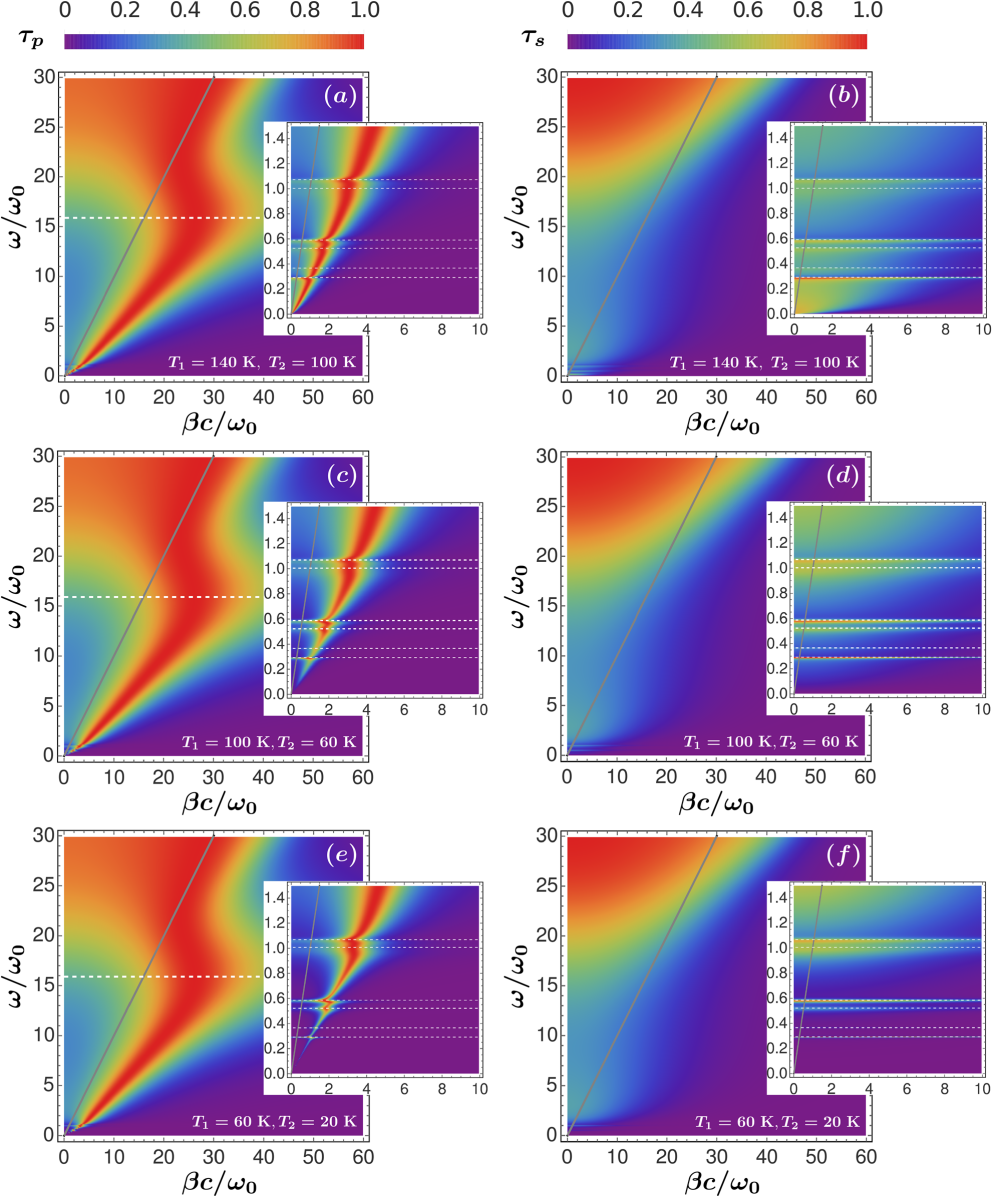
Energy transmission coefficients for P- and S-polarized waves for the three cases: (1) both plates are in the normal state (**a**,**b**); (2) One plate remains in the normal state and the other is in the superconducting phase (**c**,**d**) and; (3) Both YBCO plates are in the superconductor state (**e**,**f**). The separation between the plates is fixed at $L=50$ nm. The insets reveal the suppression of the low frequency modes caused by the superconducting transition. The dashed horizontal lines correspond to the six phonon frequencies, $\omega _{ph,l}$, and the effective plasma frequency, $\Omega _p$, of the damped mode.

To analyze the effect of the superconductive transition on the radiative heat transfer for a nanometric separation between two high-$T_c$ superconductors, we consider the three following cases: (1) the temperature of both YBCO plates is above the superconducting transition, i.e., $T_1,~T_2\geqslant T_c$ ; (2) one plate is in the normal state, $T_1 > T_c$ , and the other is in the superconducting phase, $T_2 < T_c$; and (3) both plates are in the superconducting state, $T_1,~T_2 < T_c$. Figure 4 shows the energy transmission coefficient $\tau _{p(s)}$ as a function of the normalized frequency $\omega /\omega _0$, and the dimensionless parallel component of the wave vector $\beta c/\omega _0$. The light cone lies along the gray solid line $\beta c=\omega _0$. Figure 4a,c,e are for P-polarization while b,d,f correspond to S-polarization. The separation between the plates is $L=50$ nm, and in all cases the temperature difference is kept constant, $T_1-T_2=40$ K.

For the three cases, the energy transmission coefficient $\tau _p$ displays the interplay between the several terms present in the YBCO permittivity. For $T>T_c$, in the absence of the other contributions, the Drude term would allow the excitation of a gap surface plasmon polariton (G-SPP) associated only to the free electrons. On the other hand, because of the bound electrons interband absorption, a surface mode related to the negative behavior of the MIR permitivity term would exist around $36\omega _0$. In the Supplementary Information, Fig. S2, we show the individual contributions associated to the G-SPP and the MIR electrons to the total energy transmission coefficient. For P-polarization, we see that the G-SPP is excited from DC up to $6\omega _0$. In the same frequency range also the MIR electrons contribute to $\tau _p$. The competition between these two terms results in a damped mode with an effective plasma frequency around $16~\omega _0$ approximated by the Eq. (5) (see inset of Fig. 1). The large value of $\Gamma _{mir}$ is responsible for the broad linewidth of this mode as well as for the small value of the maximum normalized wavevector, compared to the G-SPP. The surface phonon polaritons associated to the optical phonons of the YBCO hybridize with the new damped mode resulting in the Fano-like resonances we appreciate at low frequency (see insets of Fig. 4). The dashed horizontal lines represent the frequencies $\omega _{ph,l}$ of the six transverse phonons and effective plasma frequency $\Omega _{p}$.

When the temperature of at least one of the two slabs is lowered below $T_c$ the imaginary part of the permittivity suddenly drops, causing the sharp increase of the reflectivity of the corresponding YBCO slab and the excitation efficiency of the Fano-like mode decreases. This results in a frequency region of suppressed heat transfer (see Fig. 4c,d), which is more pronounced if both slabs are in the superconducting phase, as it is shown in (*e*) and (*f*), where $T_1=60$ K and $T_2=20$ K.

For all the three cases, the S-polarized energy transmission coefficient only shows frustrated phonon polaritons modes. The large $\tau _s$ values reached near the DC frequency are related to the maximum of the imaginary part of the dielectric function at zero frequency provided by the Drude term in Eq. (1), see Fig. 1b.

**Figure 5:**
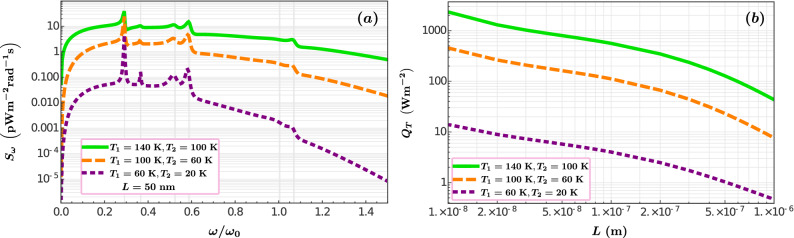
Heat flux as a function of the frequency $\omega$ (**a**) and the vacuum gap *L* (**b**) calculated for the three different cases presented in Fig. 4.

With the values of the transmission coefficients, the spectral heat flux $S_\omega$ is calculated using Eq. (7). In Fig. 5a, we plot $S_\omega$ as a function of the normalized frequency. The green solid line represents the normal-normal case, the normal-superconductor situation is depicted by the orange dashed curve, and the dotted purple line corresponds to the superconductor-superconductor system. In this case, the separation is $L=50$ nm. The superconductive transition implies a reduction of the spectral heat flux which can be as large as two orders of magnitude when both plates are in the superconducting phase. Figure 5b shows the total heat flux $Q_T$ as a function of the separation, *L*, for the same sets of temperatures used in panel a. It reveals that for a fix value of the vacuum gap *L*, between 10 and 1000 nm, the decrease of the NFRHT due to the superconductivity effect decreases two orders of magnitude when both YBCO plates transit to the superconductor state. We can expect that the reduction of the NFRHT happens even in the case of two plates separated by a few nanometers.

**Figure 6:**
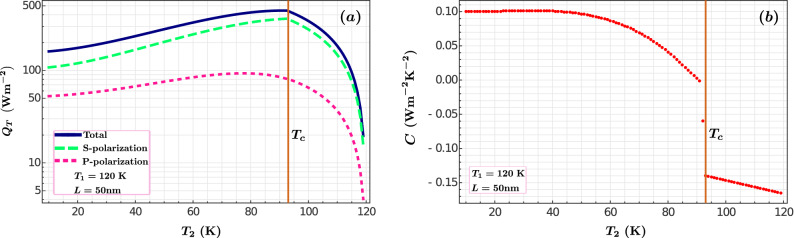
Total heat flux $Q_T$ between two YBCO plates separated by $L=50$ nm (**a**). The plate with temperature $T_2$ transits from normal to superconducting phase while the other plate remains in the normal state, $T_1>T_c$. Derivative of the heat flux $C=(1/T_2)dQ_T/dT_2$ as a function of the temperature $T_2$ (**b**).

In the results so far presented the temperature difference between the plates was kept constant, i.e., $T_1-T_2=40$ K. To understand the effect of temperature difference we fixed the temperature of one plate at $T_1=120$ K and varied the temperature of the second plate $T_2$ between 120 and 10 K. The separation between the plates was kept fixed at $L=50$ nm. In Fig. 6a the total heat transfer as a function of the temperature of the second plate is shown. As the temperature of the second plate decreases, we observe a notable increase in the heat flux $Q_T$ until the critical temperature is reached. At this point, there is a sharp slope change with the consequent reduction of the heat transfer. Recall that below the critical temperature the plate becomes more reflective and there is less energy transmission into the plate. The different curves show the contributions for P and S polarizations and the total (P+S). Most of the contribution to the total heat comes from the S-polarization, this stresses the metallic character of the YBCO provided by the Drude term in the dielectric function. As indicated in Ref.^59^, the heat flux between two metallic media separated by a distance of nanometers is principally governed by S-polarized waves. P-polarization becomes dominant only at very short distances, at which the spatial dispersion effects could manifest^59^. This is beyond the scope of our model. The contribution of P-modes becomes more significant in those materials with a plasma frequency around the far-infrared^36^. In the case of $\hbox {YBa}_2\hbox {Cu}_3\hbox {O}_{6.95}$, the effective plasma frequency $\Omega _{p}\approx 1.05$ eV falls in the near-infrared region, so that the S-contribution still dominates.

Additionally, the derivative of the heat flux with respect to the temperature defined as $C=(1/T_2)dQ_T/dT_2$ was calculated as a function of $T_2$. It is a measure of the amount of heat that plate 2 gains or looses as its temperature is modified. This is presented in Fig. 6b for a configuration in which $T_1 = 120~\hbox {K} >T_2$. For $T>T_c$, we notice that $dQ_T/dT_2$ is negative, reflecting the fact that as this plate is heated the inter-plate heat flux decreases. A relevant feature is the large discontinuity in the derivative when $T=T_c$, signaling the superconducting phase transition in slab 2. For $T<T_c$, the heat flux increases at a lower pace, until at temperatures small enough ($T\sim 50$ K) it becomes constant; hence, in this temperature regime $Q \sim T^2$. Given that only the normal charge carriers may absorb heat, this latter behavior follows straightforwardly from the two-fluid model since, according to Eq. (4), in the superconducting state $n_n(T)/n(0)=T^2/T_c^2$. We show in Supplementary Fig. S3 that qualitatively similar results are obtained for different values of $T_1$.

## Conclusions

We presented a detailed calculation and analysis of the near-field radiative heat transfer between two slabs of a high-temperature superconductor, YBCO, for different temperatures ranging from above to below the critical temperature, $T_c$. When both plates are in the normal state, the energy transmission coefficient shows that P-polarized waves’ contribution to the NFRHT is due to guided damped modes resulting from the mutual screening between free and mid-infrared electrons. At low-frequencies, the heat transfer is enhanced by Fano-like resonances arising from the hybridization of the highly damped modes with the surface phonons-polaritons. On the other hand, S-polarized frustrated phonon polaritons are also present in this frequency range. When one of the slabs is superconducting, its reflectivity increases at low frequencies, and several modes can no longer be excited for both polarizations. This decreases the total heat flux between the slabs. Finally, when both plates are superconductors, a two-order of magnitude suppression of the total NFRHT is predicted.

The anisotropic calculation demonstrates that the heat flux is weakly affected by the *c*-axis contribution (supplementary Fig. S4). As a result, the spectral heat flux between anisotropic YBCO plates is practically determined by the *ab*-plane contribution. The differential calculation of the total heat keeping the temperature of one of the plates fixed, and varying the temperature of the second one, highlights two salient features of the superconductivity effect: (1) A discontinuity in the derivative of the heat flux at $T=T_c$ consistent with the superconducting phase transition, and (2) The drop-off in heat flow inherent in the decrease of the temperature gradient ($T_1-T_2$) is associated with a negative heat flux derivative, as shown in Fig. 6 for two YBCO plates in the normal state $T_1,~T_2>T_c$. By contrast, when plate-2 becomes superconductor ($T_2<T_c$) the heat flux decreases as the temperature gradient increases entailing a positive derivative. The derived results are reminiscent of liquid $^4$He in which, below $T_c$, a superfluid and a normal components coexist; however, only this latter shows viscosity and is able to absorb heat^54^. The sensitivity of our findings to the details of the permittivity of the plates suggests that high-precision radiative thermal experiments will prove useful to understand the origin of the mid-infrared response of HTSCs, as well as helping elucidate the coupling mechanisms in these materials. Furthermore, the use of high-$T_c$ films^9,10^ can add to the control of the radiative heat transfer through the hybridization and coupling of the modes between the two surfaces of the films. This will be the topic of future research.

## Supplementary information

Supplementary information
