# Supplementary material for: Near-field radiative heat transfer between high-temperature superconductors
Source: Sci Rep. 2020 Sep 30;10:16066. doi: 10.1038/s41598-020-73017-z (PMC7527961; doi:10.1038/s41598-020-73017-z)
Supplement: Supplementary file 1 — Supplementary information [file 41598_2020_73017_MOESM1_ESM.pdf]

Near-field radiative heat transfer between high-temperature superconductors

S. G. Castillo-López[1], **G. Pirruccio**[1], **C. Villarreal**[1], and **R. Esquivel Sirvent**[1,*]

[1]Instituto de Física, Universidad Nacional Autónoma de México, Apartado Postal 20-364, México 01000, México.
[*]raul@fisica.unam.mx

Supplementary Information

Real part of the YBCO dielectric function

Figure S 1 shows the individual contributions of the Drude (dashed curve), mid-infrared (dash-dotted curve), and phonons bands (dotted curve) of the real part of $YBa_2Cu_3O_{6.95}$ permittivity, Eqs. (1)-(2) of the main text. The complete dielectric functions of the normal (panel (a)) and superconducting (panels (b)-(d)) YBCO are presented with a solid line. Below the effective plasma frequency $\Omega_p = 16\ \omega_0$, the real part of the permittivity has negative values in all the cases ($\omega_0 = 10^{14}$ rad/s). The Drude-term contributes to this behavior up to 6 ω_0 while the MIR band maintains this negative value up to Ω_p.

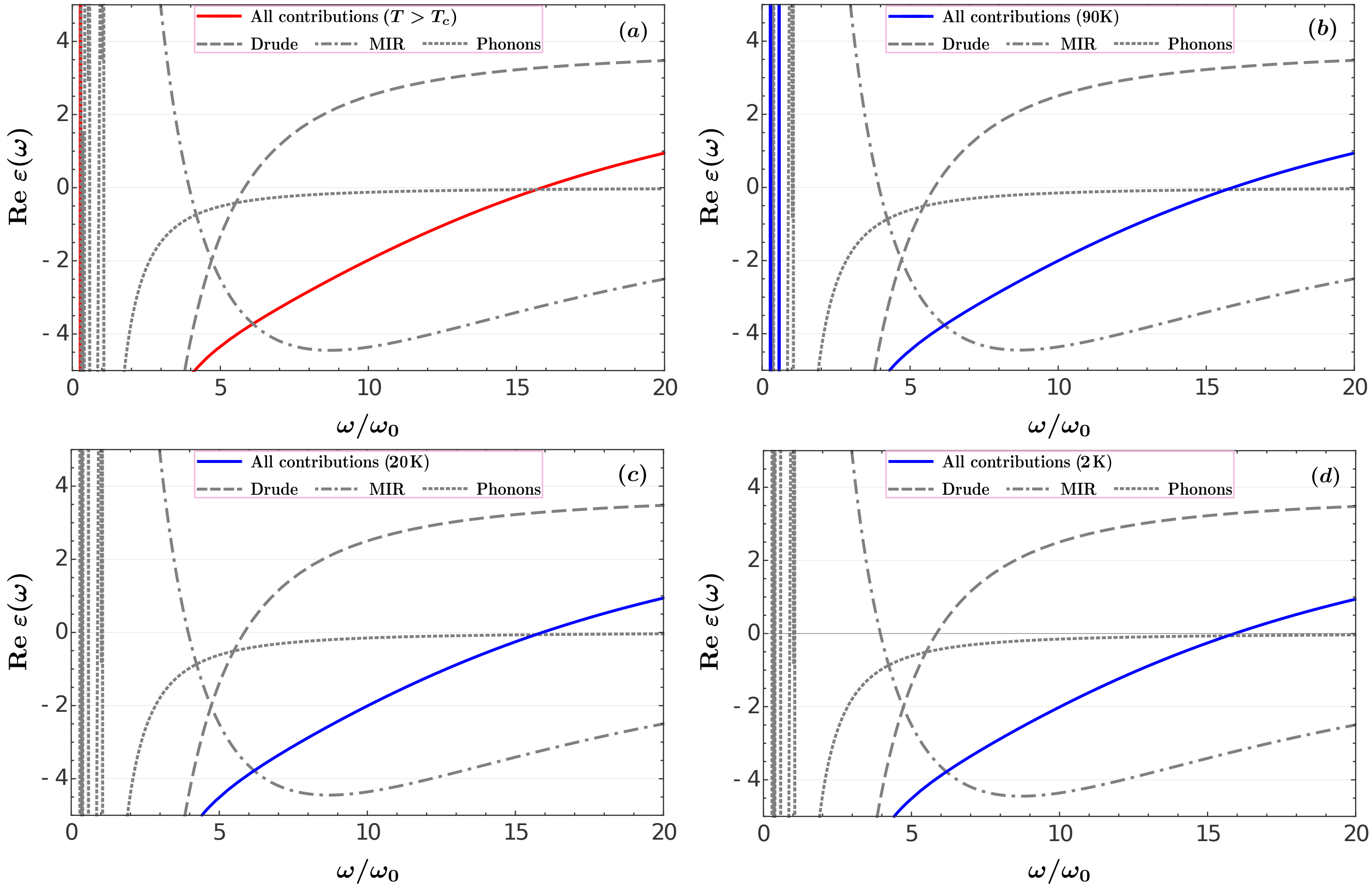

Figure S 1: Individual contributions of the Drude (dashed line), the mid-infrared (dash-dotted line) and the phonons (dotted line) bands to the real part of the normal (a) and superconducting (b)-(d) $YBa_2Cu_3O_{6.95}$ dielectric function.

Energy transmission coefficient

Figure S 2 displays the individual contributions of the free charge carriers (Drude-term) and the mid-infrared electrons to the P-polarized energy transmission coefficient, τ_p, for a cavity made of two semi-infinite $YBa_2Cu_3O_{6.95}$ plates in the normal state. Plates are separated by $L = 50$ nm. The Drude-term contribution in panel (a) shows the characteristic dispersive feature associated with a gap surface plasmon polariton (G-SPP). G-SPPs modes arise due to the coupling of the surface plasmon polaritons sustained by each YBCO plate. These modes could be excited from DC up to 6 ω_0 frequency range, where the real part of the dielectric function is negative because of the Drude contribution. In the same frequency range and extending up to higher frequencies, 65 ω_0, panel (b) shows the excitation of a spectrally broad guided mode associated with the MIR electrons. This guided mode results from the interaction of two electromagnetic modes at the surface of each YBCO plate. For large values of $\beta c/\omega_0$ the guided mode back-bends at 36 ω_0, where the MIR term of the dielectric function satisfies $\varepsilon_{mir}(\omega) + 1 = 0$.

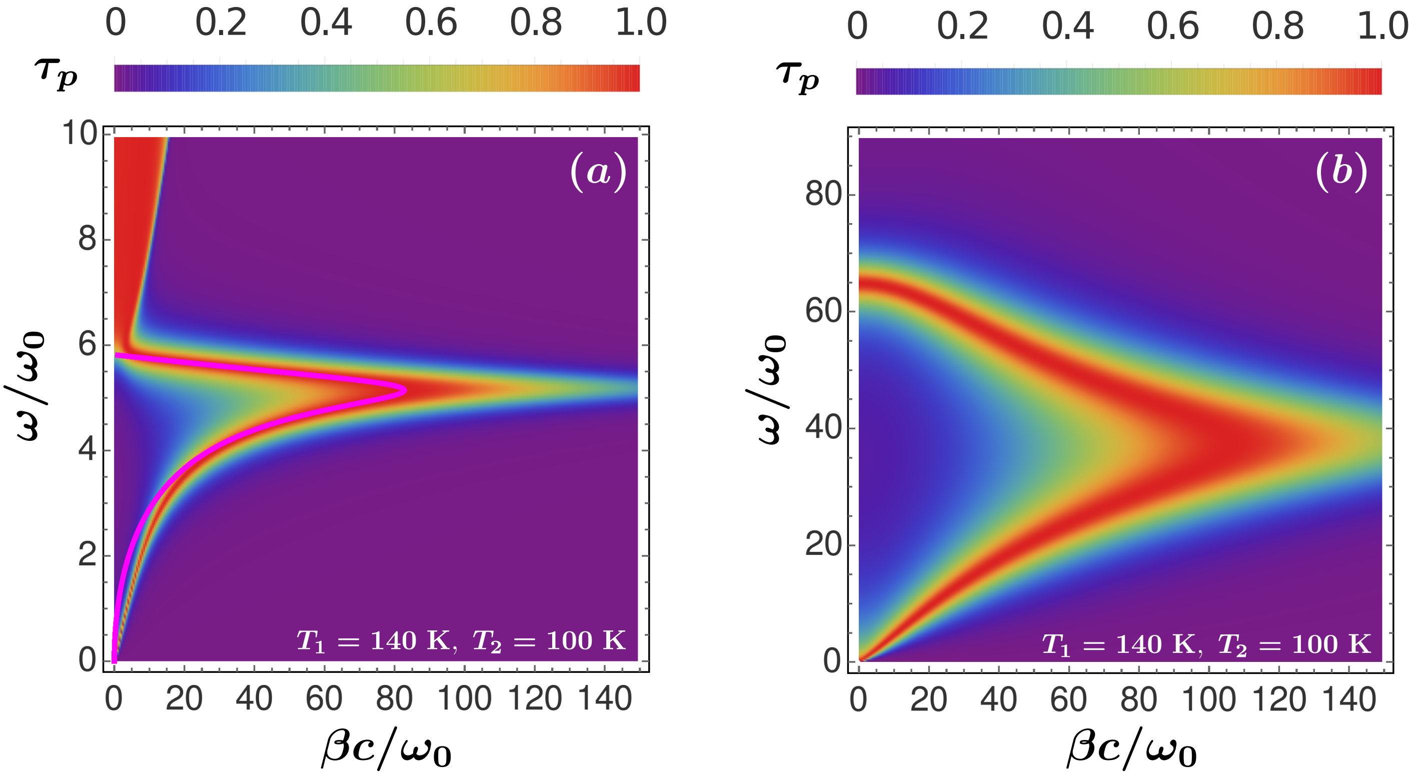

Figure S 2: Individual contributions of free charge carriers (panel (a)) and mid-infrared electrons (panel (b)) to the P-polarized energy transmission coefficient, τ_P, between two semi-infinite $YBa_2Cu_3O_{6.95}$ plates in the normal state. τ_p coefficient varying as a function of the normalized frequency ω/ω_0 and the dimensionless parallel component of the wave vector $\beta c/\omega_0$. The separation between the plates is $L = 50$ nm. Dispersion of the G-SPP is shown in panel (a) as the magenta curve.

Derivative of the heat flux

Figure S 3 displays the derivative of the heat flux $C = (1/T_2)dQ_T/dT_2$ as a function of T_2, for a configuration in which $T_1 > T_2$. Each curve is calculated for a fixed value of T_1. For the first six curves (from the top to the bottom), the plate with temperature T_2 transits from normal to superconducting phase while the other plate remains in the normal state, $T_1 > T_c$. Notice that, the size of the discontinuity at $T = T_c$ diminishes with the decrement of the temperature gradient $T_1 - T_2$. The last three curves do not exhibit the singular behavior because both plates are in the superconducting state.

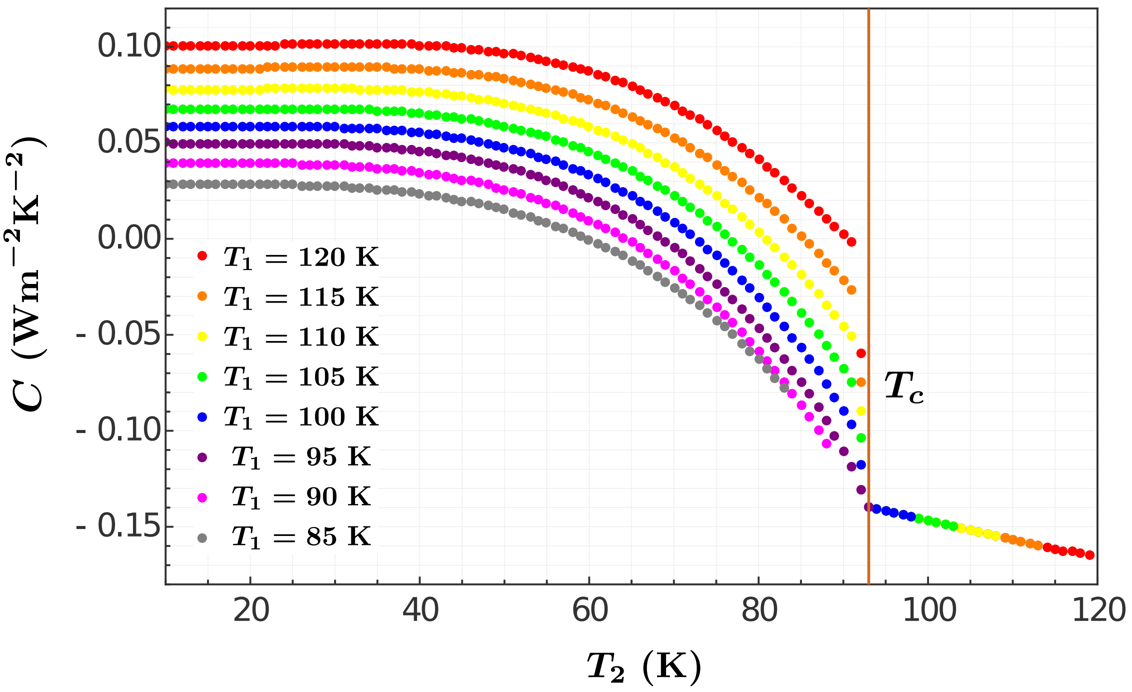

Figure S 3: Derivative of the heat flux $C = (1/T_2)dQ_T/dT_2$ for a cavity made of two $YBa_2Cu_3O_{6.95}$ plates at temperatures T_1 and T_2, with $T_1 > T_2$, as a function of T_2. Each curve is obtained for a fixed temperature T_1.

Anisotropic response

$YBa_2Cu_3O_{7-\delta}$ is an anisotropic material whose optical response on the ab-plane has been extensively studied [1, 2, 3] but not so much the response along the c-axis, see [4, 5]. A consensus exists on the electronic coherent transport along the YBCO ab-plane for temperatures below the critical T_c, which directly depends on the oxygen deficit δ. However, for underdoped compounds ($\delta = 0.2 \rightarrow 0.5$) it is not clear so far whether electron pairs condensation takes place along the c-axis too [4]. For the optimally doped $YBa_2Cu_3O_{6.95}$, there is evidence of coherent transport not only on the ab-plane but also along the c-axis [4]. Then, in the framework of a two-fluid model, the dielectric response of the $YBa_2Cu_3O_{6.95}$ along the c-axis could be described using a dielectric function similar to that of the ab-plane (Eqs. (2)-(3) in the paper),

$$\varepsilon_{n,c}(\omega) = \varepsilon_\infty - \frac{\omega_{pc}^2}{\omega^2 + i\gamma_{0c}\omega} - \frac{\Omega_{mir}^2}{\omega^2 - \omega_{mir}^2 + i\Gamma_{mir}\omega} - \sum_{l=1}^{5} \frac{\Omega_{ph,l}^2}{\omega^2 - \omega_{ph,l}^2 + i\gamma_{ph,l}\omega}, \qquad \text{for} \quad T \geq T_c. \tag{1}$$

Just above the critical temperature, the free charge carriers parameters are $\omega_{pc} = 4.65\ \omega_0$ and $\gamma_{0c} = 0.91\ \omega_0$ [4]. For the c-axis dielectric function, we also consider the mid-infrared contribution but with an oscillator strength $\Omega_{mir} = 20\ \omega_0$. The specific parameter values of the corresponding optical phonons can be found in Ref. [5].

In the superconducting phase, the dielectric function becomes,

$$\varepsilon_{s,c}(\omega) = \varepsilon_\infty + \frac{i\pi\omega_{ps,c}^2(T)}{2\omega}\delta(\omega) - \frac{\omega_{ps,c}^2(T)}{\omega^2} - \frac{\omega_{pn,c}^2(T)}{\omega^2 + i\gamma_{0c}\omega} - \frac{\Omega_{mir}^2}{\omega^2 - \omega_{mir}^2 + i\Gamma_{mir}\omega} - \sum_{l=1}^{5} \frac{\Omega_{ph,l}^2}{\omega^2 - \omega_{ph,l}^2 + i\gamma_{ph,l}\omega}. \tag{2}$$

The plasma frequencies of condensed and normal fractions of carriers exhibit the quartic temperature dependence characteristic of the Gorter-Casimir two-fluid model [4],

$$\frac{\omega_{ps,c}^2(T)}{\omega_{pc}^2} = 1 - \left(\frac{T}{T_c}\right)^4; \qquad \frac{\omega_{pn,c}^2(T)}{\omega_{pc}^2} = \left(\frac{T}{T_c}\right)^4, \qquad \text{for} \quad T < T_c. \tag{3}$$

Modeling the $YBa_2Cu_3O_{6.95}$ as a uniaxial anisotropic material, its permittivity tensor can be written as

$$\tilde{\varepsilon} = \begin{pmatrix} \varepsilon_{ab} & 0 & 0 \\ 0 & \varepsilon_{ab} & 0 \\ 0 & 0 & \varepsilon_c \end{pmatrix}. \tag{4}$$

In-plane dielectric function ε_{ab} corresponds to Eqs. (1)-(2) of the main paper, while c-axis permittivity ε_c is given by Eqs. (1)-(2) of the Supplemental. The heat flux equations (6)-(8) of the main paper can be applied in the case of uniaxial symmetry with the previous modification of the Fresnel equations as follows [6, 7],

$$r_p^{(i)} = \frac{\varepsilon_{ab}\kappa - \kappa_p}{\varepsilon_{ab}\kappa + \kappa_p} \qquad r_s^{(i)} = \frac{\kappa - \kappa_s}{\kappa + \kappa_s}, \tag{5}$$

where

$$\kappa_p = \sqrt{\varepsilon_{ab}\ \omega^2/c^2 - \frac{\varepsilon_{ab}}{\varepsilon_c}\beta^2}, \quad \text{and} \quad \kappa_s = \sqrt{\varepsilon_{ab}\ \omega^2/c^2 - \beta^2}. \tag{6}$$

Figure S4 shows the spectral heat flux between two semi-infinite $YBa_2Cu_3O_{6.95}$ plates separated by $L = 50$ nm in three different cases: (a) the temperature of both plates is above T_c, (b) the temperature of one of them is below T_c, and (c) the temperature of both plates is below T_c. In each panel, the solid line is the spectral heat flux obtained by modeling the material with the following dielectric tensor diag(ε_{ab}, ε_{ab}, ε_{ab}), the dashed line corresponds to the case diag(ε_c ,ε_c ,ε_c), and finally the dotted line is the full anisotropic calculation using Eqs. (4)-(6). In all cases, for frequencies higher than 0.4 ω_0, the spectral heat flux corresponding to the c-axis is above the curve corresponding to the ab-plane response. This is due to the low plasma frequency $\omega_{pc} = 4.65\ \omega_0$ in the c-axis compared with that in the ab-plane $\omega_p = 11.4\ \omega_0$, thus decreasing the amount of energy that is reflected by the plates increasing their heat absorption. However, the anisotropic result demonstrates that the heat flux is weakly affected by the c-axis contribution. As a result, the spectral heat flux between anisotropic YBCO plates is practically determined by the ab-plane contribution.

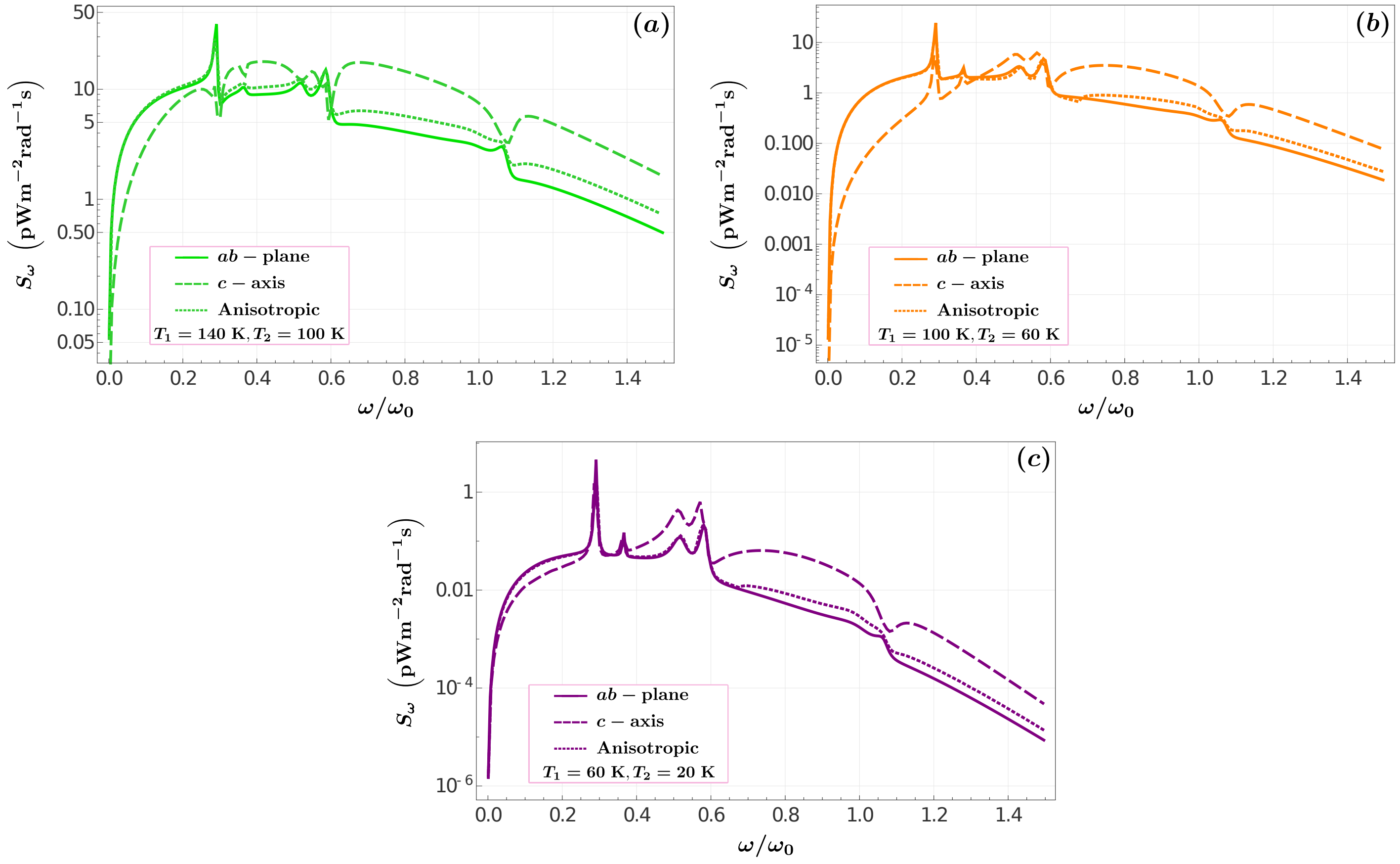

Figure S 4: Spectral heat flux between two semi-infinite $YBa_2Cu_3O_{6.95}$ plates separated by $L = 50$ nm in three different cases: (a) the temperature of both plates is above T_c, (b) the temperature of one of them is below T_c, and (c) the temperature of both plates is below T_c. In each panel, the solid line is the spectral heat flux obtained by modeling the material with the following dielectric tensor diag(ε_{ab}, ε_{ab}, ε_{ab}), the dashed-line corresponds to the case diag(ε_c ,ε_c ,ε_c), and the dotted line is the full anisotropic calculation using Eqs. (4)-(6).

References

[1] Timusk, T., *et al.* Infrared studies of ab-plane oriented oxide superconductors. *Phys. Rev. B* **38**, 6683–6688, DOI: 10.1103/ PhysRevB.38.6683 (1988).

[2] Basov, D. N. Timusk, T. Electrodynamics of high-Tc superconductors. *Rev. Mod. Phys.* **77**, 721–779, DOI: 10.1103/ RevModPhys.77.721 (2005).

[3] Bonn, D. A. et al. Far-infrared properties of ab-plane oriented YBa2Cu3O7 $- \delta$. *Phys. Rev. B* **37**, 1574–1579, DOI: 10.1103/ PhysRevB.37.1574 (1988).

[4] Homes, C. C., Timusk, T., Bonn, D. A., Liang, R., Hardy, W. N. (1995). Optical properties along the c-axis of YBa2Cu3O6+ x, for x= 0.50→ 0.95 evolution of the pseudogap. *Physica C* **254**(3-4), 265-280.

[5] Homes, C. C., Timusk, T., Bonn, D. A., Liang, R., Hardy, W. N. (1995). Optical phonons polarized along the c axis of YBa2Cu3O6+ x, for x= 0.5→ 0.95. *Can. J. Phys.* **73**(11-12), 663-675.

[6] Stinson, H. T., *et al.* (2014). Infrared nanospectroscopy and imaging of collective superfluid excitations in anisotropic superconductors. *Phys. Rev. B* **90**(1), 014502.

[7] Biehs, S. A., Ben-Abdallah, P., Rosa, F. S., Joulain, K., Greffet, J. J. (2011). Nanoscale heat flux between nanoporous materials. *Opt. Express* **19**(105), A1088-A1103.
